# Formal compliance versus functional access to diagnostics in the PhilHealth primary care benefit package: a glimpse into a rural health unit in the Philippines

**DOI:** 10.1186/s41182-026-00978-8

**Published:** 2026-05-21

**Authors:** Shogo Kanamori, Marcelyn D. Bonhaon, Yuriko Egami, Norielyn M. Evangelista, Nenita G. Marayag, Antonio F. Dela Resma Villanueva, Manami Uechi, Richard Albert J. Ramones, Mianie L. Umalco, Masami Fujita

**Affiliations:** 1 Bureau of Global Health Cooperation, Japan Institute for Health Security, 1-21-1 Toyama, Shinjuku-ku, Tokyo, 162-8655 Japan; 2 Independent Consultant, Daulan, Amduntog, Asipulo, Ifugao, Philippines; 3 Office for Health Laboratories, Department of Health, San Lazaro Compound, Santa Cruz, Manila, 1003 Philippines; 4 Health and Social Welfare Unit, Economic Research Institute for ASEAN and East Asia, 6/f Senayan II, No. 8 Jl. Asia-Afrika, Gelora Bung Karno, Senayan, Jakarta Pusat, 10270 Indonesia; 5 Local Government of Asipulo, Neduntog, Antipolo, Asipulo, Ifugao, Philippines

**Keywords:** Diagnostics, Primary health care, Universal health coverage, Philippines, PhilHealth

## Abstract

Access to essential diagnostic services is a key component of effective primary health care and universal health coverage. In the Philippines, the Philippine Health Insurance Corporation (PhilHealth) has introduced a primary care benefit package, the Yaman ng Kalusugan Program (YAKAP), which provides capitation-based payments to accredited providers to deliver a defined set of primary care services. Under this scheme, providers are required to offer a set of 15 laboratory and diagnostic tests. However, many primary care facilities, particularly rural health units (RHUs) managed by local government units (LGUs), operate with limited laboratory infrastructure and human resources. This correspondence describes a case from an RHU in the Cordillera Administrative Region (CAR) serving approximately 16,000 people. Of the 15 diagnostic tests required under the YAKAP package, nine are provided through memoranda of agreement with external laboratories located in other municipalities rather than being performed on-site. Patients referred for these diagnostic tests are required to travel approximately one to two and a half hours and incur out-of-pocket transportation costs of about 700 Philippine pesos (US$ 12) per round trip.

While such arrangements allow facilities to meet accreditation requirements, they may not ensure timely and affordable access to diagnostics for patients. Strengthening diagnostic capacity within primary care facilities is, therefore, critical. Increasing awareness among LGUs of the importance of diagnostics in PHC may encourage greater investment in laboratory services. In addition, the development of a National Essential Diagnostics List could help clarify which tests should be available at primary care facilities and guide prioritized resource allocation.

## Background

According to the World Health Organization (WHO), diagnostics include medical devices used for in vitro and in vivo determination of physiological status or the presence and characteristics of disease [1]. Access to essential diagnostic services is a critical component of effective primary health care (PHC) and universal health coverage (UHC) in low- and middle-income countries [2]. Timely diagnostic testing enables surveillance on early detection of diseases, supports clinical decision-making, and facilitates monitoring of treatment outcomes, thereby improving prognosis and enhancing patients’ quality of life and livelihood [3]. Recognizing the importance of strengthening PHC, the Philippines enacted the Universal Health Care Act in 2019 to expand access to integrated health services [4]. As part of these reforms, the Philippine Health Insurance Corporation (PhilHealth) introduced a nationwide primary care benefit package named “Yaman ng Kalusugan Program (YAKAP)”, formerly known as the Konsulta package, to strengthen primary care services through accredited health care providers [5].

Although UHC reforms and PHC strengthening initiatives have been implemented in many low- and middle-income countries, less attention has been paid to whether diagnostic services included in health insurance benefit packages are functionally accessible at the point of care, particularly in geographically remote and resource-constrained settings. In the Philippines, limited evidence exists regarding how diagnostic requirements under the YAKAP accreditation system translate into actual patient access to services at the primary care level.

The YAKAP package provides capitation-based payments to accredited providers to deliver a defined set of services, including consultations, selected medicines, and a set of laboratory and diagnostic tests [6]. Integrating diagnostic services into primary care aims to support early detection and management of common conditions and reduce unnecessary referrals to higher-level facilities.

To obtain accreditation under the YAKAP package, healthcare service providers must demonstrate the capacity to offer a specified set of diagnostic services [6]. These include 15 laboratory and diagnostic tests, such as complete blood count, urinalysis, fecalysis, fasting blood sugar, and lipid profile. By requiring these tests as part of accreditation, the YAKAP program aims to ensure that essential diagnostic services are available at the primary care level.

However, many primary care facilities in the Philippines—particularly government-run facilities—operate with limited laboratory infrastructure, equipment, and trained laboratory personnel. A substantial proportion of YAKAP-accredited providers are rural health units (RHUs) managed by local government units (LGUs), where laboratory capacity may be constrained [7]. As a result, facilities may face challenges in providing the full range of required diagnostic services on-site.

Similar challenges in access to diagnostic services at the primary care level have been reported in other low- and middle-income countries in Southeast Asia [2, 8]. However, evidence remains limited on how accreditation-based diagnostic requirements within health insurance benefit packages translate into functional access to diagnostic services at the primary care level in geographically remote settings.

### Case observation from a rural health unit

The observations presented in this correspondence are based on field visits conducted at an RHU situated in a geographically remote area of the Cordillera Administrative Region. The RHU is managed by a municipal government and serves a population of approximately 16,000 people. Semi-structured interviews were conducted by the second author, who is familiar with the local context, with the Municipal Health Officer (MHO) and Medical Laboratory Technologist (MLT) to understand the availability of diagnostic services, referral arrangements, and challenges in implementing diagnostic requirements under the YAKAP package. Information from interviews was supplemented by direct observations and review of facility-level diagnostic service arrangements.

To address the diagnostic capacity constraints, YAKAP providers may enter into memoranda of agreement (MOAs) with external laboratories or partner facilities. Through these arrangements, diagnostic tests required under the YAKAP package can be performed at another facility, while the accredited provider remains responsible for patient management. However, limited evidence exists regarding whether these referral-based arrangements ensure timely and affordable patient access to diagnostic services in geographically remote primary care settings. In particular, little is known about how reliance on external laboratories affects the practical accessibility of diagnostics for patients despite formal compliance with accreditation requirements.

In the RHU observed in this study, diagnostic services required for YAKAP accreditation are partly delivered through partnerships with other facilities in different municipalities. Of the fifteen diagnostic tests required under the YAKAP package, nine are provided through MOAs with external laboratories rather than being performed on-site at the RHU, meaning that most of the required diagnostic services are not available on-site at the facility.

Field observations and interviews identified several barriers in accessing referred diagnostic services, including long travel times, transportation costs, and dependence on referrals to facilities in other municipalities. Patients referred for these diagnostic tests must travel to partner facilities located in other municipalities at a considerable distance from the RHU. According to interviews with the MHO and MLT, patients referred for diagnostic testing typically travel between one and two and a half hours to reach partner facilities in other municipalities and incur out-of-pocket transportation costs of at least 700 Philippine pesos (US$ 12) per round trip. Although the frequency of referrals was not systematically assessed, interviewees indicated that referral-based diagnostic arrangements are routinely used for tests unavailable at the RHU.

### Implications for access to diagnostics

While MOA arrangements are a pragmatic and necessary strategy in resource-limited settings, heavy reliance on external facilities raises questions about whether accreditation requirements translate into meaningful access to diagnostic services for patients. When diagnostic tests are not available at the point of care, patients may need to travel long distances to complete recommended tests. The additional time and transportation costs may discourage some patients from completing diagnostic procedures or returning for follow-up consultations.

Fragmented service delivery across multiple facilities may also complicate coordination of care. Delays in obtaining test results and difficulties in tracking whether referred patients completed recommended diagnostics may undermine the intended benefits of integrating diagnostics into primary care services, potentially leading to delayed clinical decision-making and poorer health outcomes. Similar challenges related to limited diagnostic capacity, referral dependence, and geographic barriers have been reported in other low- and middle-income countries, including settings in Southeast Asia [2, 8]. However, this correspondence highlights an additional implementation challenge: even when facilities formally comply with accreditation requirements through referral arrangements, substantial geographic and financial barriers may still limit patients’ functional access to diagnostic services. These observations contribute to the existing literature by illustrating how health insurance accreditation requirements may not always translate into functional access to diagnostics in rural primary care settings.

### Policy recommendations

Addressing this gap requires greater attention to strengthening diagnostic capacity within primary care facilities. First, LGUs, which manage a majority of primary care facilities in the Philippines, should be further sensitized to the importance of diagnostic services in PHC. Greater awareness of the role of diagnostics in disease detection and management may encourage LGUs to allocate more financial and operational resources to laboratory services, including investments in laboratory infrastructure, equipment, and trained personnel.

Second, the development of a National Essential Diagnostics List (NEDL), which is currently underway in the Philippines, may serve as an important policy tool for strengthening diagnostic services [9]. A national diagnostics list could help clarify which diagnostic tests should be available at different levels of the health system. In particular, the Tier 1 (primary care facility) diagnostics list should be formally adopted and incorporated into the YAKAP package. This would require the development of clearer policies and implementation guidelines outlining how Tier 1 diagnostics are to be operationalized within primary care settings, including standards for on-site service provision, phased capacity-building strategies, and accountability mechanisms for compliance. Aligning YAKAP requirements with the NEDL would help ensure that accreditation standards translate into actual availability of diagnostic services at the point of care. However, the development of an NEDL alone will not automatically ensure the availability of diagnostic services or equipment at the primary care level. Effective implementation will require adequate financing, procurement mechanisms, infrastructure development, workforce strengthening, and referral system support to translate policy priorities into actual service availability.

Importantly, the effectiveness of these policy approaches will depend on how they are operationalized in practice. Previous experiences in many low- and middle-income countries have shown that policies and accreditation standards do not necessarily translate into actual service availability without adequate implementation support, financing, monitoring mechanisms, and local health system capacity. The observations from this correspondence underscore the importance of ensuring that policy reforms are accompanied by practical strategies to strengthen on-site diagnostic capacity and patient access in geographically remote settings.

In addition, further surveys are needed to assess the cost efficiency and feasibility of providing YAKAP diagnostic tests at primary care facilities. Findings from such surveys could inform diagnostic network optimization (DNO) models [10] aimed at optimizing the placement of laboratory services, specimen referral systems, and resource allocation, and could guide PhilHealth in refining the implementation of diagnostic requirements under the YAKAP package.

Similar policy approaches have been implemented in other low- and middle-income countries, including strengthening tiered laboratory networks, expanding specimen referral systems, and introducing essential diagnostics lists to guide resource allocation and service delivery [2, 9, 10]. These approaches aim to improve geographic access to diagnostics while optimizing limited health system resources. The Philippine experience described in this correspondence highlights the operational challenges that may arise when such strategies are implemented in geographically remote primary care settings.

Although this correspondence presents observations from a single facility, it highlights an important implementation challenge in strengthening diagnostics within primary care. Further research is needed to examine the extent to which YAKAP-accredited providers rely on external laboratory arrangements and to assess the implications of these arrangements for patient access to essential diagnostic services. Addressing these challenges will be critical for ensuring that health system reforms under the Universal Health Care Act translate into meaningful improvements in access to diagnostics and primary health care services in the Philippines. Furthermore, the observations in this study highlight the importance of ensuring that accreditation requirements are aligned with functional service availability at the point of care. Strengthening on-site diagnostic capacity and reducing dependence on external referrals will be essential for translating policy reforms into equitable access to diagnostics in rural and resource-constrained settings.

## Data Availability

No datasets were generated or analysed during the current study.

## Abbreviations

LGU: Local government unit
MHO: Municipal Health Officer
MLT: Medical Laboratory Technologist
MOA: Memorandum of agreement
PHC: Primary health care
PhilHealth: Philippine Health Insurance Corporation
RHU: Rural health unit
YAKAP: Yaman ng Kalusugan Program
